# Progression of Infection after Surgical CT Navigation-Assisted Aspiration Biopsy of a Vertebral Abscess

**DOI:** 10.1155/2016/8675761

**Published:** 2016-02-01

**Authors:** Vasiliki Spyropoulou, Raimunda Valaikaite, Amira Dhouib, Romain Dayer, Dimitri Ceroni

**Affiliations:** ^1^Pediatric Orthopedic Service, Geneva University Hospitals, 6 rue Willy-Donzé, 1211 Geneva 14, Switzerland; ^2^Pediatric Radiology Service, Geneva University Hospitals, 6 rue Willy-Donzé, 1211 Geneva 14, Switzerland

## Abstract

*Background Context*. Computed tomography- (CT-) guided fine-needle aspiration biopsy of the vertebral body is an important tool in the diagnostic evaluation of vertebral osteomyelitis. The procedure is considered simple to perform and it is considered a safe procedure with few complications.* Purpose*. The purpose of this study was to describe an unusual complication due to a CT-guided fine-needle aspiration biopsy of the vertebral body of L3, to better understand the relationship between surgical procedure and complication, and to reflect on how to avoid it.* Study Design/Setting*. Case report and literature review.* Methods*. The medical records, laboratory findings, and radiographic imaging studies of an 11-year-old boy, with an unusual complication due to a CT-guided fine-needle aspiration biopsy of the vertebral body of L3, were reviewed.* Results*. We report a case of vertebral osteomyelitis of L3 caused by methicillin-sensitive* Staphylococcus aureus (MSSA)*. Following a computed tomography-guided aspiration biopsy of the vertebral body of L3, vertebral osteomyelitis rapidly progressed into the vertebral body of L4 as well as the L3-L4 disk.* Conclusions*. Based on the present case, one should consider that a CT-guided fine-needle aspiration biopsy of the vertebral body may be complicated by a progression of a vertebral osteomyelitis into both the intervertebral disk and also the adjacent vertebral body.

## 1. Introduction

Vertebral osteomyelitis (VO) is an uncommon cause of back pain involving nonspecific and vague clinical features. VO starts gradually and may follow an indolent course making early diagnosis difficult [1]. Correct diagnosis of the infection is often delayed by 6 to 12 weeks due to ambiguous symptoms [2], and this delay means that VO may already have concluded before diagnosis is made. For clinically defined cases of VO the success rate of blood cultures for the identification of the causative organism ranges from 40% to 60% [3]. Many authors thus consider that computed tomography- (CT-) guided fine-needle aspiration biopsy of the vertebral body is an important tool in the diagnostic evaluation of such an infection. The procedure is considered simple to perform and could potentially be carried out early on during the course of the patient's stay in hospital, thereby enabling more prompt treatment [4]. Furthermore, it is considered a safe procedure with few complications [5]. Thus, this case report's goal was to describe an unusual complication due to a CT-guided fine-needle aspiration biopsy of the vertebral body of L3, to better understand the relationship between surgical procedure and complication, and to reflect on how to avoid it.

## 2. Case Report

**Figure 1:**
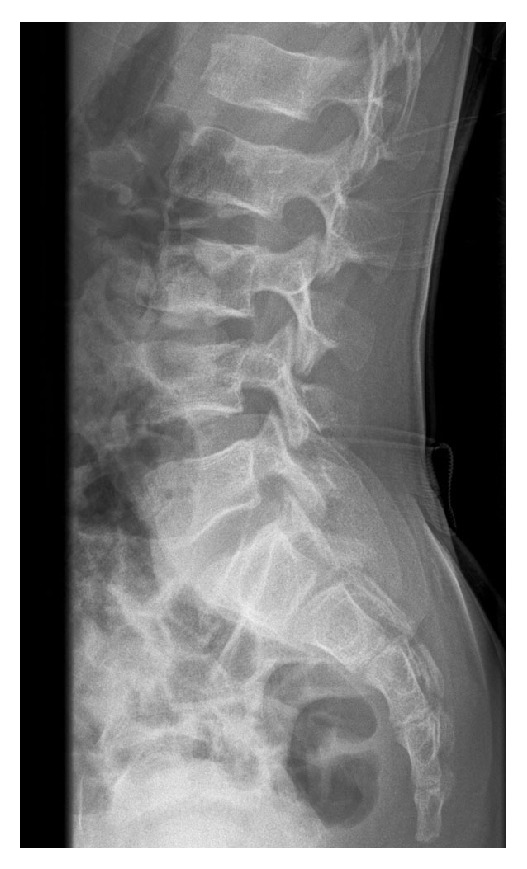
Lateral radiograph of the lumbar spine shows normal L3-L4 disk and normal endplates of L3 and L4.

**Figure 2:**
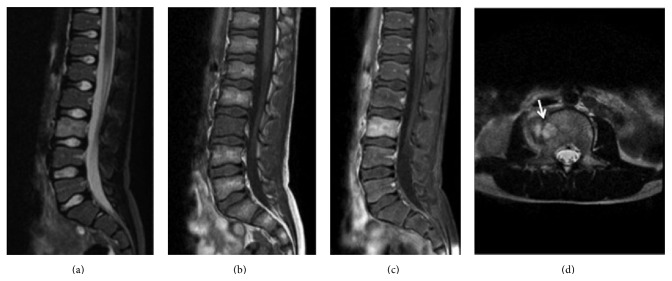
MRI scan of the lumbar spine. Sagittal spin-echo T2 weighted MR image (a) and T1 weighted MR image (b); contrast-enhanced T1 weighted image with fat saturation (c) only reveal edematous changes of the vertebral body of L3 with integrity of the L3-L4 disk. Axial Short tau inversion recovery MR image (d) shows vertebral abscess with extension to the paravertebral soft tissues (white arrow).

**Figure 3:**
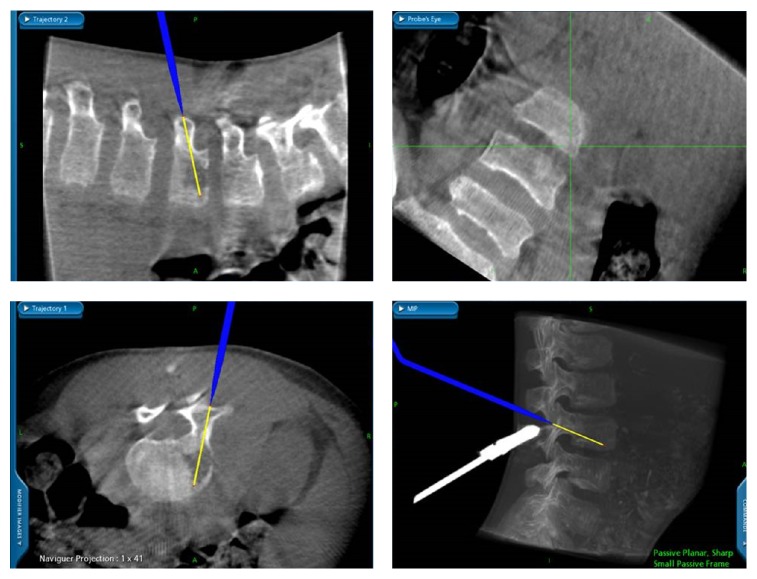
Image during CT-guided aspiration biopsy.

**Figure 4:**
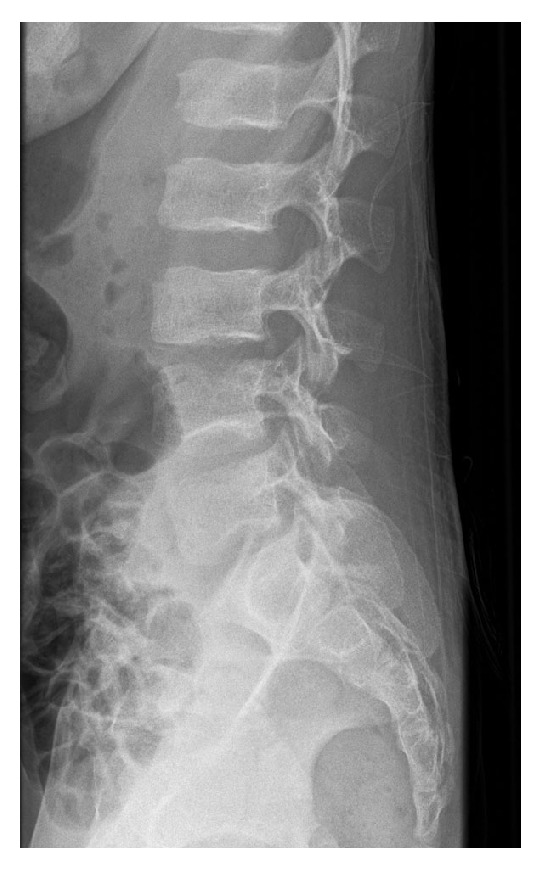
Lateral radiograph of the lumbar spine 4 days after the surgery shows L3-L4 disk space narrowing with erosion of the inferior endplate of L3 and superior endplate of L4.

**Figure 5:**
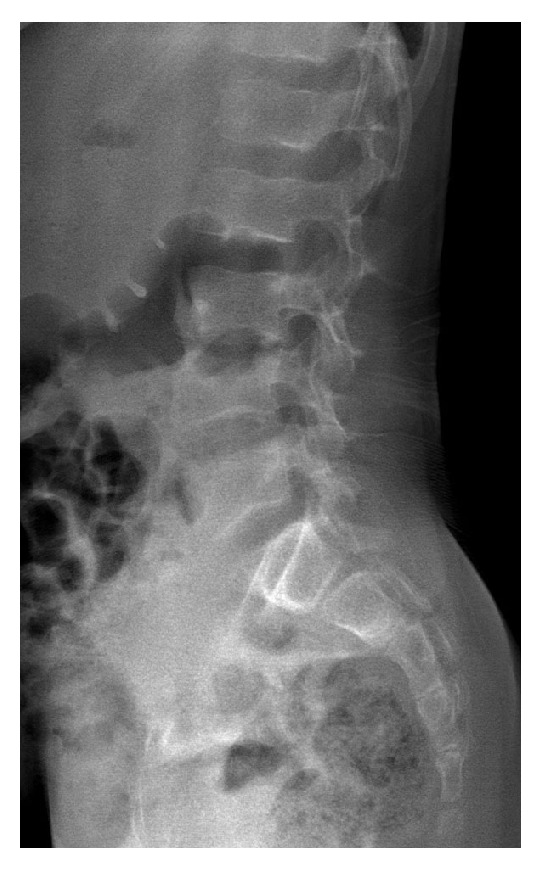
Lateral radiograph of the lumbar spine 2 months after the surgery shows complete collapse of the L3-L4 disk space with marked sclerosis, irregularities, and erosion of the inferior endplate of L3 and superior endplate of L4.

A previously healthy 11-year-old boy was admitted to our hospital with a 20-day history of low back soreness and difficulty walking and sitting up. There was no history of trauma, tooth extraction, or spinal surgery, but the patient had reported an episode of fever (39°C) within the previous 3 weeks. On examination, he had no fever, complained of lumbar soreness which was exacerbated by forward flexion of the trunk, and presented paraspinal muscle spasms. The neurological examination was otherwise normal. Laboratory tests revealed a serum WBC count of 9,800 leucocytes/mm^3^ (normal range is 4,500–13,500 leucocytes/mm^3^), a CRP (C-reactive protein) level of 35 mg/L (normal < 10 mg/L), and an ESR (erythrocyte sedimentation rate) of 48 mm/h (normal range 0–10 mm/h). Lumbar radiographs did not show any spinal lesions (Figure 1). An MRI (magnetic resonance imaging) scan revealed an abscess in the L3 vertebral body with an adjacent vertebral body hyperintensity indicative of a bone marrow oedema. There was also an extension to the soft tissues, with an intense oedema visible at the level of the right psoas (Figure 2). These findings were suggestive of a vertebral osteomyelitis without infectious intervertebral disc (IVD) damage. Because tuberculosis or a bone tumour could not be definitively ruled out, a surgical navigation-assisted aspiration biopsy of the L3 vertebral body was performed using a posterior transpedicular approach (Figure 3). The patient was stepped down to i.v. Flucloxacillin (1 g three times daily) monotherapy as soon as the aspiration biopsy was realized. Three days after admission, standard blood culture was positive for methicillin-sensitive* Staphylococcus aureus* (*MSSA*), and cultures of vertebral bone aspirate yielded growth, thus confirming* MSSA* as the pathogen. Bacteriological investigations demonstrated that the responsible strain of* MSSA* was resistant to penicillin G but sensible to all other tested antibiotics (Flucloxacillin, Gentamicin, Ciprofloxacin, Clindamycin, Erythromycin, Cotrimoxazole, Fosfomycin, Rifampicin, Tetracycline, Vancomycin, Teicoplanin, and Linezolid). Imaging studies confirmed that there was no evidence of endocarditis. Three additional sets of postoperative monitoring blood cultures were also positive, continuing up until 8 days after the surgical procedure. A standard X-ray of the lumbar column was carried out 4 days after surgery and revealed a sudden 30% decrease in the height of the disk space, together with erosions of the adjacent vertebral endplates (Figure 4). The patient went on to complete a two-week course of i.v. Flucloxacillin (1 g three times daily), followed by a four-week course of orally administered Clindamycin (30 mg/kg/j). He responded well to antibiotics and body orthosis and showed clinical and laboratory improvement durably after the end of antibiotic treatment. Nevertheless, 2 months after surgery, a new X-ray revealed a considerable progression of the initial lesion which now included the vertebral body of L4 as well as the L3-L4 disk; there had been complete collapse of the disc and major erosion of the upper vertebral endplate of L4 (Figure 5). Nevertheless, the patient no longer reported low back soreness and he was able to move freely.

## 3. Discussion

Childhood spondylodiscitis represents a continuum of infectious conditions of the spine, from discitis to vertebral osteomyelitis [6]. Usually the most common pathway of VO is a result of haematogenous seeding. VO is rare in children; its incidence rate is approximately 0.3 per 100,000 in patients younger than 20 years old [7]. Several studies have highlighted a triphasic age distribution, with varying signs and symptoms according to age [6, 8]. In this context, children with vertebral osteomyelitis tend to be older than those with discitis [6, 8–10]. The lumbar spine is the most commonly affected region [11, 12], and involvement of the posterior elements is exceptional [13].

Blood cultures should always be taken for children with vertebral osteomyelitis because they are often the only guide for selecting the appropriate antimicrobial therapy. The positive blood culture rate is greater than in spondylodiscitis but only affects from 40% to 60% of clinically defined cases [3]. The indication for more invasive procedures, such as biopsy or needle aspiration, is currently not established [14]. Many authors consider that a spinal specimen should be obtained by closed percutaneous or open surgical biopsy. The literature shows that the success rates for needle aspiration and open biopsy in identifying causative organisms range from 0% to 63% for spondylodiscitis [8, 15–17]. Using spinal specimens for patients with VO, de Lucas et al. reported that the positive culture rate reached 43% [4]. However, due to the surgical and anesthetic risks, these interventions are still not considered standard diagnostic procedures by most authors [14].

Progression of a vertebral osteomyelitis into the adjacent vertebral body and the IVD after CT-guided aspiration biopsy of a vertebral abscess has rarely been reported, especially in children [18]. In the case reported by Ziegelbein and El-Khoury, the boy was 10 years old (11 years old in our case) and he was not suffering from leucocytosis as was our patient [18]. The ESR and the CRP level were elevated, at 42 mm/h (48 mm/h in our case) and 41 mg/L (35 mg/L in our case), respectively [18]. Disk space narrowing was noted 7 days (4 days in our case) after the CT-guided aspiration biopsy of the vertebral abscess with progression (as in our case) of the infection through the adjacent vertebral endplate [18].

Why our patient sustained a strong and precocious progression of the infection into the IVD and adjacent vertebral body after a CT-guided aspiration biopsy of the vertebral body is somewhat an enigma. In order to understand it, we need to go back to the blood supply to the IVD. It must be remembered that the IVD of infants and children has a much more profuse vascular supply than adults [19]. The capillary network at the vertebral margins adjacent to the discs is denser in infants and children than in adults. Several vessels penetrate deeply into the discs of infants, whereas in most adolescents and in all adults no such vessels are present. The most acknowledged hypothesis is that VO originates from an infected microembolus in the arterial system which becomes lodged in one of the metaphyseal arteries; this results in infarction and subsequent haematogenous spread of the infection. Subsequently, spontaneous secondary discitis usually results as the VO spreads into the IVD. Hence, one can hypothesize that the vertebral aspiration biopsy probably induced an increase of the intraosseous pressure and thus facilitated the passage of septic emboli through the vessels that penetrate deeply into the disc.

Why the patient sustained a discitis when he had received antibiotics remains another perplexing element. One must nevertheless keep in mind that, for a prophylactic antibiotic to be effective, it must be present in a sufficient concentration in the IVD from the start of surgical incision and for the duration of the procedure [20–22]. Although most antibiotics penetrate vascular tissue well, there is no agreement in the literature regarding their ability to enter discs in an active form. Furthermore, the antibiotic's ability to spread through all parts of the disc is influenced not only by the vascular supply and structure of the disc (size and health), but also by the properties of the drug (size, solubility, binding, and charge) [23]. The antibiotic's charge in particular has been discussed in the literature, since the nucleus pulposus is rich in glycosaminoglycans and has a high density of negative charge [23]. Thus, it has been postulated that positively charged antibiotics (Gentamicin or Vancomycin) can enter the IVD, whereas negatively charged antibiotics (penicillin and cephalosporins) have limited [20, 24, 25] or poor penetration [26] because of repellent charges.

## 4. Conclusions

This case raises questions about the relevance and the best way of performing an aspiration biopsy in cases of VO. Clinicians should consider a vertebral aspiration biopsy for patients with negative blood cultures, especially when tuberculosis or bone tumours can be discounted. Computed tomography-guided vertebral drainage is a straightforward and accurate technique for diagnosing VO in such cases [10]. However, it would be advisable to perform an aspiration biopsy of the soft tissue abscess (a paravertebral injection) rather than osseous ponction in order to avoid the possible transfer of septic emboli through the vessels that penetrate deeply into the disc. Prophylactic or therapeutic antibiotics should be chosen not only for their ability to deal with the suspected microorganism, but also with regard to their ability to penetrate the IVD. Finally, antibiotic exposure before biopsy does not have a negative impact on pathogen recovery, and thus it is suggested that antibiotic treatment should start before undertaking biopsies in children with suspected VO.
